# Controlling for body size leads to inferential biases in the biological sciences

**DOI:** 10.1002/evl3.151

**Published:** 2019-12-19

**Authors:** Björn Rogell, Damian K. Dowling, Arild Husby

**Affiliations:** ^1^ Department of Zoology Stockholm University Svante Arrhenius väg 18 Stockholm Sweden; ^2^ Department of Aquatic Resources, Institute of Freshwater Research Swedish University of Agricultural Sciences Drottningholm 17893 Sweden; ^3^ School of Biological Sciences Monash University Clayton Victoria 3800 Australia; ^4^ Centre for Biodiversity Dynamics Norwegian University of Science and Technology 7491 Trondheim Norway; ^5^ Evolutionary Biology, Department of Ecology and Genetics Uppsala University 75236 Uppsala Sweden

**Keywords:** Allometry, brain size, collinearity, comparative methods, sexual dimorphism

## Abstract

Many traits correlate with body size. Studies that seek to uncover the ecological factors that drive evolutionary responses in traits typically examine these responses relative to associated changes in body size using multiple regression analysis. However, it is not well appreciated that in the presence of strongly correlated variables, the partial (i.e., relative) regression coefficients often change sign compared to the original coefficients. Such sign reversals are difficult to interpret in a biologically meaningful way, and could lead to erroneous evolutionary inferences if the true mechanism underlying the sign reversal differed from the proposed mechanism. Here, we use simulations to demonstrate that sign reversal occurs over a wide range of parameter values common in the biological sciences. Further, as a case‐in‐point, we review the literature on brain size evolution; a field that explores how ecological traits relate to the evolution of relative brain size (brain size relative to body size). We find that most studies show sign reversals and thus that the inferences of many studies in this field may be inconclusive. Finally, we propose some approaches to mitigating this issue.

**Impact summary**

Multiple regression analysis is a commonly used method in biology to identify the ecological factors that drive evolutionary responses in key morphological or life‐history traits that are independent of associated changes in body size. However, when these traits are strongly correlated to body size, then the partial (i.e., relative) regression coefficients can spuriously change sign compared to the original coefficients, something that could lead to erroneous evolutionary inferences. We used simulations to demonstrate that these sign reversal occurs over a wide range of parameter values common in the biological sciences. To examine the occurrence of such sign changes in the empirical literature, we reviewed studies that have investigated the putative role of various ecological variables in driving the evolution of relative brain size. Given that body size and brain size are usually strongly correlated, studies on the evolution of brain size provide an excellent case study to investigate the challenges associated with drawing evolutionary inferences in the presence of strong collinearity. We find that, within the field of brain size evolution, most studies show sign reversals, and thus inferences from these studies may be inconclusive or incorrect. Our results suggest that the methods routinely used by biologists to account for covariation between focal traits and body size may be prone to producing misleading estimates that result in widespread inferential errors with respect to the underpinning evolutionary processes.

Body size varies widely across species (Blueweiss et al. 1978), and has pervasive effects on nearly all biological traits, from the size of other body parts (Lande 1979; Huxley 2011), to key life history traits such as fecundity (Honěk 1993) and lifespan (Peters 1986; Speakman 2005). As such, strong allometric relationships often exist between body size and other traits, and these relationships constitute an intrinsic characteristic of many biological datasets. Due to the large influence of body size, an important goal in biology is therefore to understand the evolutionary drivers that affect trait variation (be they morphological, physiological, or life‐history traits) independently of the effects of body size. This can be achieved by including body size as a covariate in multiple regression models (Lande and Arnold 1983), and thereby estimating the relative relationships in the form of partial regression coefficients. These partial regression coefficients indicate the change in the response variable given a unit change in the corresponding predictor, keeping all other variables in the model constant (Lande and Arnold 1983). Henceforth, we will discuss models that aim at estimating a relative relationship between a trait in strong allometry with body size (“trait of interest”), and an ecological variable (hereafter “selective agent”), while controlling for body size.

The estimated relative effects can be evolutionarily important and have a biological interpretation. Indeed, artificial selection experiments where selection has been applied on relative trait values (hence experimentally controlling for body size), have shown that relative trait values can respond to selection; for example, relative gonopodium length in mosquitofish (Booksmythe et al. 2016), relative wing size in *Bicyclys* butterflies (Frankino et al. 2007), and relative brain size (Kotrschal et al. 2013), and fin size (Egset et al. 2012) in guppies. In all these examples, changes of the relative trait values were associated with no, or only minor changes in body size. Similar results have also been obtained from comparative analyses (e.g., Kruska 1988; Deaner and Nunn 1999; Shattuck and Williams 2010; Healy et al. 2014). In accordance with these examples, the suggested causation in comparative studies, which control for body size statistically rather than experimentally, is often that selection acts on the relative values of the trait of interest (i.e., size of the trait relative to body size).

In the presence of strong allometric relationships among traits (collinearity), however, drawing inferences about selection acting on relative trait values is not straightforward. While collinearity has been argued to cause statistical issues such as increased uncertainty and biased parameter estimates (García‐Berthou 2001; Freckleton 2011; Dormann et al. 2013), a largely ignored consequence of collinearity is sign reversal (or sign flipping; Friedman and Wall 2005). This occurs when the regression coefficient between the trait of interest and selective agent is positive (or negative) when not controlling for body size, whereas it becomes negative (or positive) when body size is included in the statistical model. That is, the sign of the regression coefficient changes when estimating the relative relationship between the trait of interest and the selective agent when including body size in the model.

Such sign changes are thus caused by differences in the correlation between the selective agent and the trait of interest and the correlation between the selective agent and body size. The occurrence and frequency of sign changes are therefore dependent on the differences in these correlations and are likely to manifest particularly when collinearity is high (Fig. 1; Friedman and Wall 2005). As a consequence, estimates derived from multiple regression models can be challenging to interpret when variables are highly correlated (i.e., when there is strong collinearity).

**Figure 1:**
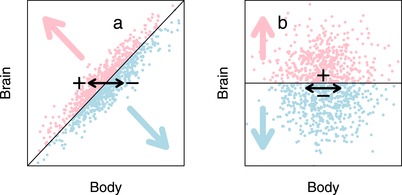
A schematic illustration of how evolutionary changes can result in large and small values of a trait of interest, such as, for example, brain size. In a scenario with allometry (a), selection on body size can shift brain size from small (blue dots) to large (pink dots) brain size, something that cannot occur in the absence of an allometric relationship (b). The pink and blue dots here represent large and small brains, respectively, the pink and blue arrows represent areas of segregation on the inferential values (big vs. small brain), which are relative to body size in (a) but not in (b).

While there are many biological scenarios in which the factors contributing to the expression of variable of interest are likely to exhibit high collinearity with each other. In particular, strong allometric relationships often exist between body size and other traits, for example, in comparative studies (Fig. 2). If a selective agent induces divergent selection on body size, it is unlikely that traits that are in strong allometry to body size would evolve completely independently from body size, but would instead be likely follow the same direction as the evolution of body size. However, since traits often differ in their evolutionary rates, evolutionary lags are expected between traits whereby one trait may exhibit a lower‐than‐expected response than a second correlated trait in response to a selective agent (Riska 1991; Hansen and Bartoszek 2012; Smaers et al. 2012, but see Deaner and Nunn 1999). Hence, if the selective agent is positively related to body size, the absolute correlation between the trait of interest (for example, brain size) and the selective agent is also likely positive. However, due to the more rapid divergence in body size compared to brain size, the correlation between brain size and the selective agent is likely to be negative on a scale relative to body size (i.e., for similarly sized brains, the relative brain size will be determined by the size of the body, not the brain). Under such conditions, the selective agent will correlate with relative brain size even if there is no direct selection on brain size (Fig. 2). As a result, concluding that selection has been operating on relative brain size can be questioned when there are concordant changes in body size (Smaers et al. 2012).

**Figure 2:**
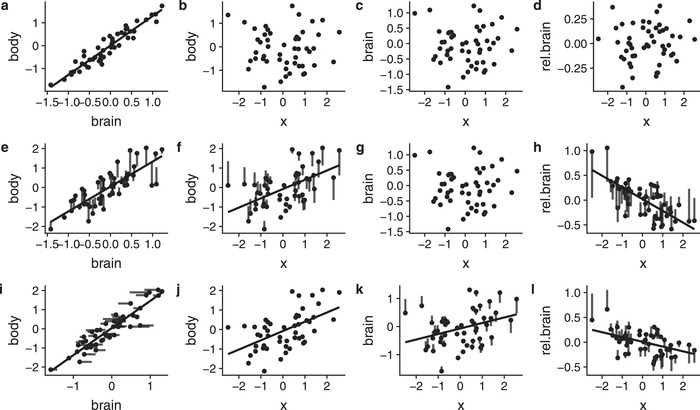
A stepwise schematic illustration of how evolutionary changes can drive partial associations between a selective agent (x) and relative brain size. The three steps depicted are (a–d) the ancestral stage, (e–h) evolutionary changes in body size but not brain size, and (i–l) how brain size may follow changes in body size due to its allometric relationship. More specifically, (a)–(d) represent a number of species with a strong allometry between brain and body size (a), but without a relationship between body size and x (b), brain and x (c), as well as relative brain size and x (d). In (e–h) divergent selection on body size, driven by x, causes a positive correlation between body size and x (f), and strongly negative relationship between x and relative brain size (h), despite there being no relationship between absolute brain size and x (g). The correlation between x and relative brain size is here driven by the direct effect of x on body, rather than, brain size. (i–l) However, the allometric relationship between brain and body size makes it unlikely for such independent evolution of body size and brain size, but due to evolutionary lags, the compensation is likely to be only partial, causing a positive association between x and brain size (k), and a negative association between relative brain size and x (l). The lines associated to the points, indicate the change in the position of the points compared to the previous state. The presence of a regression line indicates a significant relationship.

To investigate the extent of sign reversal of the partial regression coefficients in the empirical literature, we examined studies that have focused on the ecological and social drivers of brain size variation in metazoans (particularly in birds and mammals). This field has embraced the comparative approach as a critical tool in the study of brain‐size ecology, and inspired a wave of research studies over the past two decades that report a plethora of variables associated with relative brain size (Eisenberg and Wilson 1978; Møller et al. 2005; Benson‐Amram et al. 2016; Sayol et al. 2016). However, these comparative studies have also at times been criticized on the basis that relative brain size can be a poor proxy for the cognitive abilities of an organism (Healy and Rowe 2007; Chittka and Niven 2009). A further, hitherto unappreciated, complication in such studies is that if sign reversal is common, this could potentially explain the large number of studies reporting evolutionary shifts in relative brain size to different ecological selection pressures.

Here, we explore the occurrence of sign changes in the empirical literature and discuss the evolutionary scenarios that could yield these patterns. In addition to reviewing the literature to assess the frequency of sign reversal, we also used simulations to explore the range of allometric relationships where sign reversals occur, and to test if sign reversals are prone to particular types of statistical model or if they occur across the different models that are commonly used in ecological and evolutionary studies.

## Materials and methods

### LITERATURE SURVEY

We carried out a literature survey to examine the extent of sign reversal in comparative studies on brain size evolution. We searched Google Scholar using the keywords: “relative brain size,” “evolution,” “comparative analysis,” and “ecology,” which generated ∼900 studies. To enable comparisons between studies, we only included studies were the datasets were publicly available and that we could re‐analyze to estimate standardized coefficients (by scaling all continuous variables to a mean of zero and a unit standard deviation prior to analysis; see supplementary material for an overview of studies included). In our reanalysis of these datasets, we included the selective agent as the response variable because that provides the partial regression coefficients for both body size and brain size with the selective agent, allowing for a direct assessment of possible sign changes. In cases where the partial association between relative brain size and the selective agent were dependent on a factorial variable in the original study (e.g., if the effect only occurred in a specific sex or mating system), the relationship was analyzed within each factor level. If the selective agent was a factorial variable with two levels, we assessed the sign of the partial regression coefficients using a linear model with the arcsine square root transformed proportion as response. We excluded selective agents that were factorial variables with more than two levels (n = 21 variables, from three studies). All analyses were performed on the species level, and if there were several entries for each species, we calculated the species‐specific mean.

We stress that our aim was not to replicate the results from each study, but rather to examine how common sign reversals of the partial regression coefficients are in comparative studies on brain size evolution. We therefore limited our choice of studies to those in which a phylogenetically uncontrolled analysis gave the same qualitative result as a phylogenetically controlled analysis in terms of the signs of the partial regression coefficients (as assessed from the values reported in the published studies). Given the constraints above, we were able to include 129 estimates from 34 different studies where we could estimate the correlations between brain size, body size (both on log‐scale), and the selective agent, as well as the partial regression coefficients between brain size and the selective agent, and body size and the selective agent (see Supporting Information).

From each study, we recorded whether the signs of the two partial associations between brain size and selective agent and body size and selective agent changed when both were included in the same model (i.e., a sign reversal) compared to when fitted singly. Finally, we examined whether there were any effects of sample size, the absolute correlations between selective agent and brain size and body size, and the correlation between body size and brain size on the propensity to obtain sign changes. This was done in a generalized linear mixed model using lme4 in R that included study identity as a random effect. Note that we fitted each of these terms (sample size, absolute correlation between body size and brain size and that between selection agent and brain size and body size) separately and thus only evaluate their overall and not relative effect.

### SIMULATIONS TO EXAMINE FREQUENCY OF ANTAGONISTIC PARTIAL REGRESSION COEFFICIENTS

A partial regression coefficient between trait of interest (brain size) and the selective agent can be obtained by modeling either the trait of interest or selective agent as the response variable, including the other variable as a covariate together with body size. From a collinearity perspective, these models can therefore be divided into (1) models that are characterized by strong collinearity among the explanatory variables (the selective agent is added as a response, while body size and brain size are added as explanatory variables; and (2) models that are characterized by strong collinearity between the response and one of the explanatory variables. Here, brain size is added as a response variable while body size and the selective agent are added as explanatory variables. We used simulations to assess if sign reversal occurs at similar values of correlations (brain size and body size, brain size and selective agent, body size and selective agent) across these two model categories. The standardized partial regression coefficient *β*_*x*_ (the partial regression coefficient for trait of interest on the selective agent) for a multiple regression given by $Y=\alpha +\beta_{1}X+\beta_{2}Z+\varepsilon$, can be calculated from the three possible correlations between *Z*, *X*, and *Y*, as $\beta_{x}=\frac{\rho_{x,y}-\rho_{x,z}*\rho_{z,y}}{1-\rho_{x,z}^{2}}$. Sign shifts occur due to the subtraction in the numerator, and, if the correlation between *Z* and *Y* is close to 1, then the sign of the partial regression coefficient will be determined by minor differences between the correlations *ρ*_*y,x*_ and *ρ*_*y,z*_. If Z is body size, the same subtraction in the numerator occurs regardless of whether the trait of interest or the selective agent is modeled as a response (Y) or predictor (X). Consequently, we expected strong correlations between trait of interest and body size to lead to sign changes regardless of modeling approach, and we further explored this using simulations.

We simulated data using the R package *mvtnorm* (Genz and Bretz 2009), setting the correlation between body size and the selective agent constant at *r* = 0.5, and the correlation between brain size and the selective agent varied between *r* = 0.35 and 0.65, with a step size of 0.001 (150 draws). Sign changes are caused by differences between the correlations selective agent and trait of interest and that between selective agent and body size, in combination with strong correlations between brain size and body size. Given that evolutionary allometric relationships (and their associated correlations) are usually very strong (Huxley 2011), we varied the correlation between brain size and body size between *r* = 0.85 and 0.98 with a step size of 0.001 (130 draws). Thus, in total we simulated 130 × 150 = 19,500 data sets.

In addition to simulating continuous variables, we also examined a larger set of models that can be categorized under two main types, including models on residual values, models where one of the focal traits was assessed as a factorial variable, and models where the response is a factorial variable (see Supporting Information). Each simulated data set was used to examine the effect on the fitted regression coefficients using ordinary least squares regression models. For each model type and simulation run, we extracted the correlation between body size and the selective agent as well as the partial regression coefficients between the selective agent and brain size. We could thereby identify the type of models and the set of correlations (brain–body, brain–trait of interest, body–trait of interest) where controlling for body size causes sign reversals of the estimated regression coefficients, and compare these across model types.

## Results

### WIDESPREAD INFERENTIAL BIAS IN COMPARATIVE STUDIES ON EVOLUTION OF BRAIN SIZE

**Figure 3:**
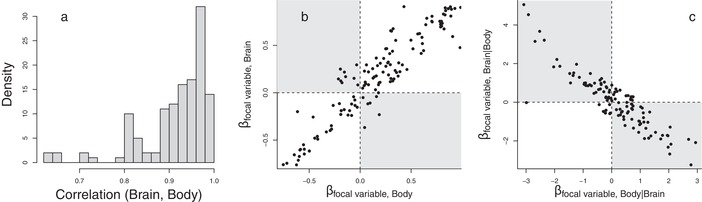
(a) The distribution of the correlation coefficients between brain size and body size on the log scale from the empirical data. (b) The univariate regression coefficients of brain size on the focal variable plotted against univariate regression coefficients of body size on the focal variable. Note that the correlations between the focal variable and the two traits are almost always of the same sign (very few observations can be found in the gray quadrants). (c) The partial regression coefficients for brain size plotted against the partial regression coefficient for body size. Note that almost all observations are in the region of antagonistic effects (gray quadrants). In (b) and (c), there were some extreme outliers (all following the general pattern described in each respective plot), and hence, we plotted the data that falls in the 95% confidence intervals.

As expected, across studies, the correlation between brain size and body size was very high, with a median correlation coefficient of 0.938 (range: 0.629–0.997; Fig. 3). When we examined the sign of the different correlations from these studies, we found that the correlation between the selective agent and absolute brain size was positive (66% of the cases, binomial test from 50%: *P* < 0.001) and of substantial magnitude (mean: 0.40, SD: 0.27). Similarly, the correlation between the selective agent and absolute body size was positive in 64% of studies (binomial test from 50%, test statistic, *P *< 0.001) and of a similar magnitude (mean: 0.38, SD: 0.28). The correlations between absolute body size and the selective agent tended to be in the same direction as the correlations between absolute brain size and the selective agent (87% of cases, binomial test to 54% [i.e., the joint probability of obtaining the same sign], *P* < 0.001). This demonstrates that the correlation between the selective agent and absolute brain size and between the selective agent and absolute body size are significantly more often in the same direction.

In strong contrast, the great majority (81%) of the 129 estimates produced opposite signs of the partial regression coefficient for the effects of relative body size (i.e., relative to brain size) and relative brain size on the selective agent (Fig. 3, exact binomial test to 46% [i.e., the joint probability of not obtaining the same sign], *P* < 0.001). Thus, if the relationship between relative body size and the selective agent was in one direction, the relationship between relative brain size and the selective agent showed an effect in the opposite direction (Fig. 3). When restricting the test to include only partial associations between brain size and the focal trait that were statistically significant, the proportion of estimates with sign reversal increased to 91% (70 estimates, exact binomial test to 0.46% [i.e., the joint probability of not obtaining the same sign in the subset], *P* < 0.001).

The combination of mostly concordant signs for the relationships between absolute brain size and absolute body size to the selective agent, and mostly discordant signs for the partial relationships, implies that either relative brain size or relative body size changes the sign of the relationship with the selective agent compared to the absolute trait values. The sign reversal occurred more frequently for the association between relative body size and the selective agent (66%) than between relative brain size and the selective agent (34%, exact binomial test to 50%; *P* = 0.004). When further assessing the potential correlates of sign changes in a generalized linear mixed model, accounting for study ID, we found that sign changes occur more often for lower absolute values of correlations between selective agent and brain size (estimate = −4.5, *z* = –3.6, *P *< 0.001), and body size (estimate = –4.8, *z* = –3.3, *P* = 0.001), but were not affected by sample size or the correlation between brain and body size (*P *> 0.2). Importantly, and in all analysis, the intercept term was highly significant (estimate > 1.7, *P *< 0.001), indicating that sign changes were more frequent than expected.

### SIMULATION OF REGRESSION COEFFICIENTS

**Figure 4:**
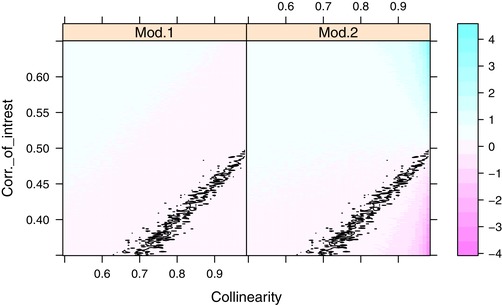
A heat map of the magnitude of the partial regression coefficients between brain size and the selective agent while controlling for body size. The correlation between body size and the selective agent is set to 0.5. The correlation between brain size and body size (Collinearity) is represented on the *x*‐axis. The correlation between brain size and the selective agent (“Corr of interest”) is represented on the *y*‐axis. All correlations are on absolute scale, not partial. In the first panel (model 1, denoted “Mod.1”), the highest collinearity is between one explanatory variable (body size) and the response (brain size), whereas in the second panel (model 2, denoted “Mod. 2”) the highest collinearity is between the explanatory variables brain size and body size. The black lines within each panel indicate where sign reversal of the partial regression coefficient of brain size and the selective agent occurs. Note: (1) that sign reversal occurs at similar values of the correlations in the two panels, but that the partial effects are stronger when the collinearity is among explanatory variables (“mod 2”); and (2) due to the skew of how extreme the regression coefficients are in the two plots, the shift from positive to negative occurs within the pink area.

The simulations showed that the set of correlations (brain size and body size, brain size and trait of interest, and body size and trait of interest) where the different statistical models produced sign reversal of the partial regression coefficients (i.e., where both possible univariate regression coefficients were positive and one of the partial regression coefficients negative) was large and within a biologically relevant range. Sign reversal happened whenever the correlation between brain size and body size was lower than the product of the correlations between brain size and body size, and selective agent and body size (i.e., when |*Corr*_brain size, selective agent_|< |*Corr*_body, brain size_ × *Corr*_body, selective agent_|, Fig. 4) and occurred at very similar points for the different statistical models (Fig. 4).

## Discussion

Body size covaries with a large number of morphological, behavioral, and life‐history traits. While evolutionary divergences in the absolute values of such traits may be driven by divergent selection on body size, understanding the selective forces that operate on traits independent of body size is of key interest. The standard practice when controlling for allometric relationships with body size, is to include body size as a covariate in the statistical model, resulting in relative effects of the two traits (reviewed in McCoy et al. 2006). These relative values are deviations from an allometric relationship and therefore depend on evolutionary change in both brain size as well as body size. However, of the 34 studies we reviewed that statistically controlled for body size, only five clearly tested for associations between the selective agents and body size (Shultz and Finlayson 2010; Carrete and Tella 2011; Maklakov et al. 2011; Fitzpatrick et al. 2012; Sayol et al. 2016).

Findings from different artificial selection studies, where selection was on relative values of traits and only resulted in minor responses in body size (Frankino et al. 2007; Egset et al. 2012; Kotrschal et al. 2013; Booksmythe et al. 2016), have suggested that the size of these traits may evolve independently of body size. Furthermore, within‐species allometries for birds and mammals can be more shallow in slope than between‐species allometries, suggesting that evolutionary changes should be able to occur without corresponding shifts in body size (Tsuboi et al. 2018). Yet, across a broad range of studies and species, we found strong and concordant effects of the selective agent on both absolute brain size and body size; 87% of the univariate regression coefficients between the selective agent and body size, and between the selective agent and brain size, were of the same sign. However, when brain size and body size were analyzed as predictors in a multiple regression setting, 81% of the models produced partial regression coefficients with antagonistic signs. This demonstrates that such models are unable to disentangle effects of brain size relative to body size, and suggests that the changes are not necessarily caused by selection on relative brain size, independent of body size. Thus, our results indicate that while brain size and body size may to some extent evolve independently, many of the associations between relative brain size and various ecological factors reported in the literature cannot be statistically disentangled from concomitant associations between body size and the ecological factors.

Inferences from partial regression coefficients under strong collinearity have been criticized, given that under strong collinearity, even minor deviations in the correlations between the selective agent and body size and the selective agent and trait of interest will manifest as sign reversal in the partial regression coefficients (Friedman and Wall 2005). While we cannot infer whether the main axis of variation that determines the association is due to changes in brain size or in body size, our analysis clearly shows that the statistical models currently used by biologists, do not achieve their purported goal of controlling for body size. This is a significant obstacle when it comes to the reliability of biological inferences made using these statistical models. Moreover, collinearity is often discussed in comparative studies, yet is almost exclusively seen as an issue that relates to the inclusion of two or more highly correlated explanatory variables (Sokal and Rohlf 1994). However, the occurrence of sign reversals induced by strong correlations was similar across models where the strongest correlation is between the response and an explanatory variable and models where the strongest correlation is between two explanatory variables (Fig. 4; Supporting Information). In practice this means that, while the partial regression coefficient between the selective agent and body size is not estimated in models using brain size as response, and while these models do not have any “formal” collinearity among predictors, the partial regression coefficient between the selective agent and brain size is still equally sensitive to sign changes.

### IMPLICATIONS FOR COMPARATIVE STUDIES OF BRAIN SIZE

Most of the studies we reviewed concluded there is ecological selection on relative brain size (e.g., Isler and Schaik 2009; Navarrete et al. 2011; Benson‐Amram et al. 2016), and do not consider the potential effects of selection acting on body size leading to a correlated change in brain size as an alternative mechanism mediating the association between relative brain size and the selective agent. While selection acting directly on relative values may seem unlikely, it is possible that the evolution of larger relative brain sizes is attained through a combination of both decreases in body size and increases in brain size in response to a selective agent. Although perhaps unlikely, this could yield the surplus of antagonistic sign changes that we have observed in our study. However, the biological relevance associated with variation in relative brain size has been questioned on the basis of methodological issues associated with measuring both brain size and cognitive performance (Healy and Rowe 2007), as well as on the basis that neural functionality of the brain may not scale to its size (Chittka and Niven 2009). It therefore seems reasonable that the strong antagonistic pattern of the partial regression coefficients we observe could also result from other scenarios than selection on relative brain size.

We have shown that the regression coefficient that will reverse its sign is the one with the weakest absolute correlation to the selective agent and that sign reversals occurred more frequently for body size than for brain size. This indicates that brain size generally has a stronger correlation to the selective agents and may seem to indicate that selection on brain size is primary target with correlated changes in body size. However, an alternative explanation could lie in differences in the ontogenetic development of these two traits. As the brain develops early during ontogeny (Riska and Atchley 1985), a selective agent affecting mainly postnatal growth (as seen for example in secondary size dimorphism) may correlate more strongly to adult body size than to adult brain size. Conversely, if an evolutionary factor is dependent on prenatal growth (as seen for example in maternal effects), it may correlate more strongly to brain size than to body size, simply because the adult body size will be a composite measure of pre‐ and postnatal growth.

Body size is, moreover, tightly linked to life‐histories and is likely to be sensitive to environmental effects (Rowiński and Rogell 2017). Indeed, brain size is often more canalized than body size across the sexes (Fitzpatrick et al. 2012), and body size frequently has a higher evolutionary rate than brain size (Gonzalez‐Voyer et al. 2009; Fitzpatrick et al. 2012; Smaers et al. 2012; García‐Peña et al. 2013). Furthermore, allometric slopes across species are generally steeper than allometries within species in birds and mammals (Tsuboi et al. 2018), and under such conditions selection on body size is likely to cause a deviation from an evolutionary allometry. It seems likely therefore that selection on body size may, at least partly, underlie the reported associations between a selective agent and relative brain size. In accordance with the hypothesis that these effects stem from divergences in body size, in combination with a strong allometric relationship, the inclusion of additional explanatory factors should easily change the directionality of the effects, given that these additional factors explain parts of the residual variation in body size. The partial relationships should hence be quite volatile in their nature and dependent on other factors included in the models. Indeed, the addition of other explanatory variables have in some cases changed inferences (Dechmann and Safi 2009; DeCasien et al. 2017), suggesting that the reported effect of relative brain size may not drive the reported relationships.

### IMPLICATIONS FOR OTHER BIOLOGICAL DISCIPLINES

Comparative studies on brain size evolution are, of course, not the only studies where issues brought about by strong intertrait correlations will arise. Several other biological disciplines routinely rely on relative values to make inferences on how variables relate to each other. The issues that we describe above are therefore likely to be of concern in any study where collinearity is present. Legitimate questions may therefore be asked of whether the relative values are of biological relevance. Importantly, such effects are likely to occur at any level where there is systematic divergence in body size, this being at the species, population, or individual level. For example, analyses on rates of metabolism and growth, may also benefit from a focus on how the covariates included in their analyses change the inferences of the results. Most obviously, the problem of sign reversal applies to other comparative studies that control for body size, such as comparative studies of relative testes size, which have been important in understanding the general trajectories of reproductive trait evolution under sexual selection (Lüpold and Fitzpatrick 2015). Another example where relative values are often used is in cell biology and biochemistry, where abundances of different metabolites are highly correlated both to each other and to variation in the amount of tissue or cells used in the assays (Baris et al. 2017). While we cannot make any claims about how common this problem is in other fields, we note that such studies share similar characteristics to those that create sign reversals in comparative studies on brain size evolution.

### GUIDELINES FOR FUTURE RESEARCH

It is difficult to draw biologically reliable inferences in the presence of strong collinearity, but we nevertheless suggest the following strategy. First, it is useful to compare estimates of the rate of evolutionary change in body size and the trait of interest to clarify how selection acts on body size compared to the trait of interest; a pursuit that can aid the interpretation of the partial regression coefficients. While such analyses are highly informative, and possible to employ when a molecular phylogeny is available, they have only rarely been used (Gonzalez‐Voyer et al. 2009; Fitzpatrick et al. 2012; García‐Peña et al. 2013). Second, one obvious way to avoid collinearity will be to carefully choose study systems where there is only weak association between body size and the selective agent or between the trait of interest and body size. This is likely to be difficult, but some studies within or across populations may meet these criteria, as well as some systems where there are large divergences in life‐histories without correlated changes in body size (e.g., Eckerström‐Liedholm et al. 2017). Finally, artificial selection experiments have successfully been used to select on trait values relative to body size (Frankino et al. 2007; Egset et al. 2012; Kotrschal et al. 2013; Booksmythe et al. 2016), and these therefore provide an excellent mechanism for assessing short‐term evolutionary trajectories of the trait of interest and body size to selection, as well as their putative links to ecological variables.

It is further important that studies also report body size changes and carefully inspect for sign reversals (e.g., Husby and Husby 2014). Given the large number of statistical models that are unable to deal with collinearity, further development of statistical methods that can handle such data should be a priority pursuit for future research. In the case of studies on brain size, it could be promising to use ancestral state reconstruction to identify and compare taxonomic groups that are diverged in brain size, but not in body size (Smaers et al. 2012).

In conclusion, we have demonstrated that strong correlations between brain and body size have resulted in widespread sign reversal of the partial regression coefficients in comparative studies examining the evolution of relative brain size. This creates substantial difficulties in drawing accurate biological inferences from such studies. We also show that the parameter space where sign reversals of the partial regression coefficients occur is large, especially when collinearity is strong. In such cases, which are common in comparative studies on brain size evolution, we argue that the partial regression coefficients for brain size cannot be interpreted as independent of the effects of body size. Thus, it is necessary to ask when is a relative measure of a trait (controlling for body size) important, and when does it lead to misinterpretation? The current norm in evolutionary ecological studies of controlling for body size, in the absence of a functional understanding of how the trait of interest and body size each respond to a given selection pressure, can generate a substantial risk of drawing erroneous conclusions.

## AUTHORS CONTRIBUTIONS

The study was conceived and designed by B.R. and A.H. B.R. collected data, carried out simulations, analyzed the data, and wrote the first draft. B.R., D.K.D., and A.H. contributed to the interpretation of analyses, writing of the subsequent drafts, and revisions of the manuscript.

Associate Editor: A. Goswami

## Supporting information


**Figure S1**.
**Table S1**. Data from Husby & Husby 2013.
**Table S2**. Included studies.

Supporting Information

Supporting Information
