# Supplementary material for: Controlling for body size leads to inferential biases in the biological sciences
Source: Evol Lett. 2019 Dec 19;4(1):73–82. doi: 10.1002/evl3.151 (PMC7006466; doi:10.1002/evl3.151)
Supplement: Supplementary file 1 — Figure S1. Table S1. Data from Husby & Husby 2013. Table S2. Included studies. [file EVL3-4-73-s001.pdf]

Supplementary information

Simulations

We used simulations to examine the parameter space where sign reversal in regression coefficients happen. To achieve this, we simulated a range of co-linearities that represent the strength of the allometric relationship among two focal variables and body size. More specifically, we simulated a situation where brain size is strongly associated with body size and where the selective agent had a varying association with both body size and brain size. We used the R package *mvtnorm* to generate the multivariate correlational space (Genz and Bretz, 2009). The correlation between body size and the selective agent was set constant at $r = 0.5$, and the correlations between brain size and the selective agent varied between $r = 0.35$ and 0.5, with a step size of 0.01 (151 draws). In the case the one of the selective agent was a factorial variable, we again simulated a trivariate normal distribution as described above but now trait2 followed an underlying determinant distribution of a Bernoulli variable (a latent distribution) where the individual level probabilities were set to $1/(1+e^{-x})$, where x is the score on the simulated latent distribution. This is the inverse of the logit function, which is a commonly used linear predictor for binomial models. The latent distribution of the factorial selective agent is sampled with a mean of 0, giving a 0.5 probability for both levels of the trait. The correlation between the simulated trivariate distributions were identical to the simulations in which the selective agent was continuous.

Different types of models evaluated for continuous variables of interest.

Mod 1. Brain ~ selective agent + Body; highest collinearity expected between the response (brain) and one of the predictors (body).

Mod 2. selective agent ~Brain+Body; highest collinearity expected in the regression matrix between (brain) and (body).

Mod 3. Residuals(Brain~Body)~ selective agent ; response is the residual values from a regression with high collinearity between the response (brain) and the predictor (body).

Mod 4. Residuals(Brain~Body)~Residuals(selective agent ~Body); response is the residual values from a regression with high collinearity between the response (brain) and the predictor (body).

Mod 5. selective agent ~Residuals(Brain~Body); highest collinearity expected in the regression matrix between (brain) and (body).

Different types of models evaluated for factorial variables of interest

Mod 6. Brain~ selective agent (factorial) + Body

Mod 7. Residuals(Brain~Body)~ selective agent (factorial)

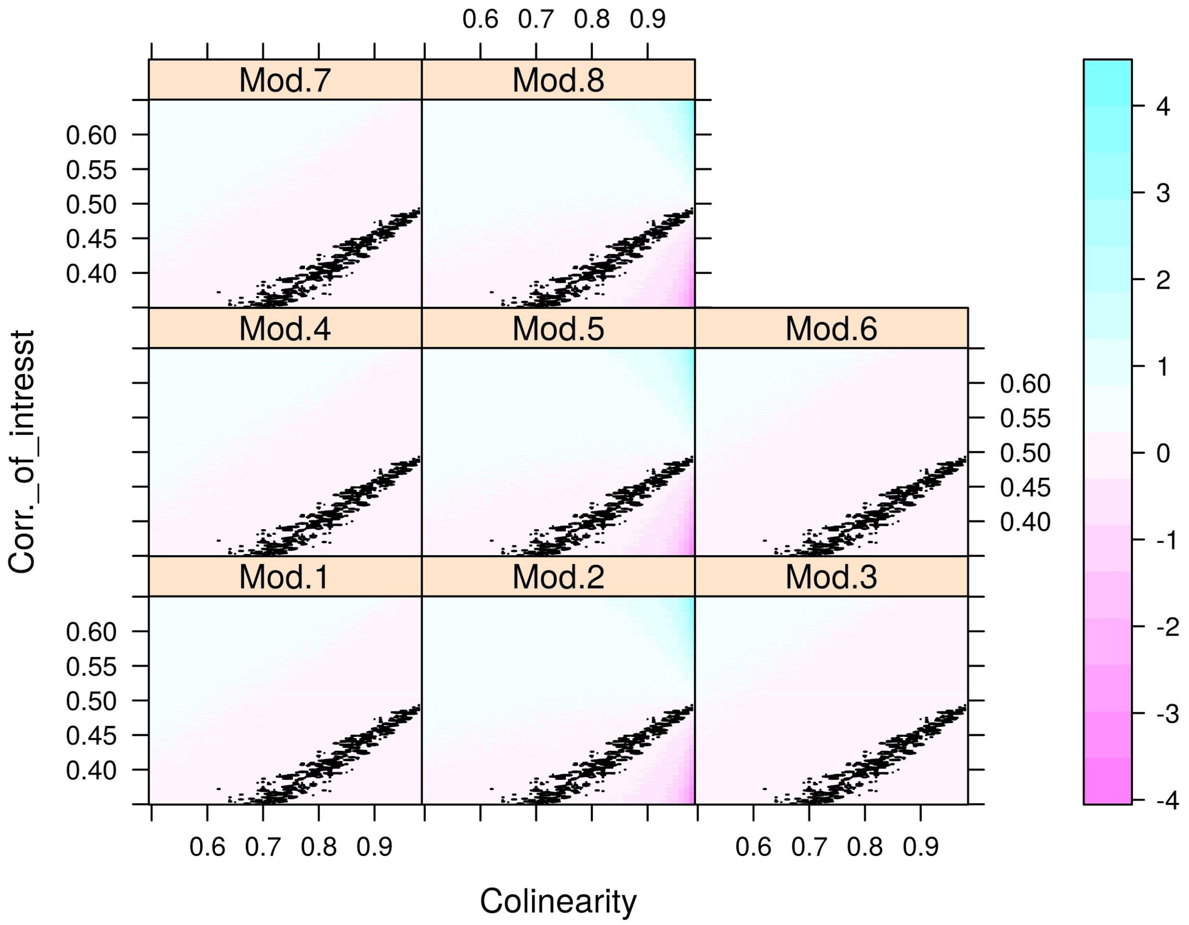

Supplementary figure 1

Heat maps of the partial regression coefficients between brain size and the selective agent while controlling for body size. On the x-axis is the collinearity between brain size and body size. On the y-axis is the correlation between brain size and the selective agent. In the panels (mod 1, mod 4, mod 7, mod 3 & mod 6) the highest collinearity is between one explanatory variable (body size) and the response (brain size), whereas in panels (mod 2, mod 5 & mod 8) the highest collinearity is between the explanatory variables brain size and body size. The lines indicate where sign reversal occurs. Note that sign reversal occurs at similar parameter settings in the two panels, but that the partial effects are stronger when the collinearity is among explanatory variables.

A hypothetical example

If a selective agent imposes divergent selection on body size, the body sizes may diverge relatively more than a trait of interest, for example brain size. This may cause deviations from the allometric relationship between trait of interest and body size, where those organisms driven by the selective agent to a small body size will have a larger relative size than the trait of interest, than those driven to a large body size (Smears et al. 2012). Under such conditions the selective agent will correlate to the relative size of the trait of interest, without any direct selection on the trait of interest (main manuscript Fig 2). Inferences of selection on relative values of trait of interest can therefore be questioned when there are concordant changes to body size.

A hypothetical example could be penguin species occurring in either polar and subtropical regions, where living in a polar region induces selection for a larger body size, with larger deposits of insulating fat tissue, as adaptations to cope with low temperatures, but no direct selection on brain size. Due to the strong allometry between body size and brain size, the polar penguins will have both larger bodies and brains. Fat content will therefore be positively correlated to both absolute brain size and body size. However, if the evolutionary potential for change is larger in body size than in brain size, as is commonly found, this could result in that the larger polar penguins may have a absolute brain size that is smaller than expected from their brain-body size allometry. As a consequence, the relative brain size of the penguins will correlate negatively with fat content, and a researcher may in this case draw the conclusion that fat tissue is in a energetic trade-off to brain size. The relationship between brain size and fat content will in this case change sign depending on if brain size is studied on a absolute scale, or a scale relative to body size. In congruence with a biological reality (evolutionary lags), but not

with the specific inference of that fat content evolve as a trade-off with relative brain size, such scenarios will be apparent as sign changes when covariates are added to a model (Friedman and Wall 2005).

An example of how to check for sign reversals

The following example is taken from Husby & Husby (2013) Interspecific analysis of vehicle avoidance behavior in birds, Behavioural Ecology. This study tested how brain size influence vehicle avoidance behavior in 11 species of European birds, and found that species with a larger relative brain size flew away from the road more often than species with a small brain size.

Table S1: Data from Husby & Husby 2013. Body is the mean body size in grams, Brain is the mean brain size in grams, "Crossed" is the number of birds that flew over the road as the vehicle passed, "Away" is the number of birds that flew away from the vehicle. "Asin.sqrt.prop" is the arcsine square root transformed proportion of birds that flew away from the vehicle.

Species	Body (g)	Brain (g)	Crossed	Away	asin.sqrt.prop
Corvus corone	479.78	8.472	165	495	1.0472
Corvus monedula	214.39	4.84	49	213	1.1236
Pica pica	204.51	5.526	166	492	1.0446
Larus canus	360.05	3.8	61	68	0.8125
Emberiza citrinella	28.65	0.822	112	152	0.8614
Fringilla coelebs	21.4	0.81	43	71	0.9095
Motacilla alba	21.11	0.598	263	392	0.8845
Sturnus vulgaris	82.59	1.925	53	134	1.0094
Passer domesticus	27.7	0.97	39	92	0.9937
Turdus iliacus	65.2	1.215	58	52	0.7581
Turdus pilaris	99.8	1.9	243	380	0.8963

Testing the correlations among traits reveal a strong correlation between log brain size and log body size (cor=0.97), and that the correlations between the likelihood of flying away from the

vehicle, (asin.prop), correlated positively with both log body size and log brain size. However, the correlation was stronger to brain size (cor=0.55) than to body size (cor=0.35).

```
> with(dat, cor(cbind(brain=log10(Brain), body=log10(Body), asin.prop)))
              brain      body  asin.prop
brain     1.0000000 0.9652809  0.551913
body      0.9652809 1.0000000  0.349229
asin.prop 0.5519130 0.3492290  1.000000
```

Testing the effect of body size, and brain size, in univariate regressions, on the likelihood of flying away from the vehicle, (asin.prop), indicate non-significant and positive regression coefficients of both brain and body size.

Brain size:

```
> summary(lm(asin.prop~log10(Brain), dat))

Call:
lm(formula = asin.prop ~ log10(Brain), data = dat)

Residuals:
     Min       1Q   Median       3Q      Max
-0.17349 -0.03106  0.02507  0.05141  0.12098

Coefficients:
```

```
            Estimate Std. Error t value Pr(>|t|)
(Intercept)  0.89465    0.03746  23.885 1.89e-09 ***
log10(Brain)  0.15762    0.07938   1.986   0.0784 .
---
Signif. codes:  0 '***' 0.001 '**' 0.01 '*' 0.05 '.' 0.1 ' ' 1

Residual standard error: 0.09837 on 9 degrees of freedom
Multiple R-squared:  0.3046,  Adjusted R-squared:  0.2273
F-statistic: 3.942 on 1 and 9 DF,  p-value: 0.07836
```

Body size:

```
> summary(lm(asin.prop~log10(Body), dat))

Call:
lm(formula = asin.prop ~ log10(Body), data = dat)

Residuals:
     Min       1Q   Median       3Q      Max
-0.17727 -0.04580  0.01611  0.07198  0.15147

Coefficients:
            Estimate Std. Error t value Pr(>|t|)
(Intercept)  0.78869    0.13943   5.657 0.000311 ***
log10(Body)  0.07868    0.07037   1.118 0.292486
```

```
---
Signif. codes:  0 '***' 0.001 '**' 0.01 '*' 0.05 '.' 0.1 ' ' 1

Residual standard error: 0.1105 on 9 degrees of freedom
Multiple R-squared:  0.122,	Adjusted R-squared:  0.0244
F-statistic:  1.25 on 1 and 9 DF,  p-value: 0.2925
```

However, when both body size and brain size are included in the same model, a different pattern emerge, where brain size have a strongly significant effect, and body size has a strongly negative effect on the likelihood of flying away from a vehicle. Here a sign shift has occurred, where body size has changed its sign in a multiple regression. Note that the regression coefficients are of a much larger magnitude in the multiple regression, and that the R-squared (the proportion of variance explained by the model), is in the multiple regression model, is larger than the sum of the R-squared values of the independent regressions.

```
> summary(lm(asin.prop~log10(Brain)+log10(Body), dat))

Call:
lm(formula = asin.prop ~ log10(Brain) + log10(Body), data = dat)

Residuals:
      Min        1Q    Median        3Q       Max 
-0.069601 -0.037425 -0.006565  0.036890  0.073577 
```

```
Coefficients:
            Estimate Std. Error t value Pr(>|t|)
(Intercept)   1.8468     0.2163   8.538 2.72e-05 ***
log10(Brain)  0.8991     0.1736   5.178 0.000845 ***
log10(Body)  -0.6059     0.1370  -4.424 0.002215 **
---
Signif. codes:  0 '***' 0.001 '**' 0.01 '*' 0.05 '.' 0.1 ' ' 1

Residual standard error: 0.0562 on 8 degrees of freedom
Multiple R-squared:  0.7982,  Adjusted R-squared:  0.7478
F-statistic: 15.82 on 2 and 8 DF,  p-value: 0.001658

>
```

Table S2: Included studies

Title	Authours	Year	Journal
Acrobatic Courtship Display Coevolves with Brain Size in Manakins (Pipridae)	Lindsay WR, Houck JT, Giuliano CE, Day LB.	2015	Brain Behav Evol.
Both social and ecological factors predict ungulate brain size	Shultz S, Dunbar R.I.M	2005	Proc Biol Sci.
Brain size and the expression of pheomelanin-based colour in birds	Galván I, Møller AP	2011	J Evol Ecol
Brain size as a driver of avian escape strategy	Samia DSM, Møller AP, Blumstein DT	2015	Scentific reports
Brain size evolution in pipefishes and seahorses: the role of feeding ecology, life history and sexual selection	Tsuboi M, Lim ACO, Ooi BL, Yip MY, Chon WC, Ahnesjö I, Kolm N	2017	J Evol Biol.
Brain size predicts problem-solving ability in mammalian carnivores	Benson-Amrama S, Dantzer B, Strickere G, Swanson EM, Holekampe KE	2015	Proc Natl Acad Sci
Brain size, life history, and metabolism at the marsupial placental dichotomy	Weisbeckera V, Goswamic A	2010	Proc Natl Acad Sci
Brains and the city: big-brained passerine birds succeed in urban environments	Maklakov A.A, Immler S, Gonzalez-Voyer A, Rönn J, Kolm N	2011	Biol Lett
Brief Communication_ Seasonality of diet composition is related to brain size in New World Monkeys	van Woerden JT, van Schaik CP, Isler K.	2014	Am J Phys Anthropol.
Coevolving avian eye size and brain size in relation to prey capture and nocturnality	Garamszegi LZ, Møller AP, Erritzøe J.	2002	Proc Biol Sci.
Comparative support for the expensive tissue hypothesis: Big brains are correlated with smaller gut and greater parental investment in Lake Tanganyika cichlids	Tsuboi M, Husby A, Kotrschal A, Hayward A, Buechel SD, Zidar J, Løvlie H, Kolm N.	2015	Evolution
Covariation between brain size and immunity in birds: implications for brain size evolution	Møller AP, Erritzøe J, Garamszegi LZ.	2005	J Evol Biol.
Do smart birds stress less_ An interspecific relationship between brain size and corticosterone levels	Lendvai AZ, Bokony V, Angelier F, Chastel O, Sol D.	2013	Proc Biol Sci.
Effects of Seasonality on Brain Size Evolution_ Evidence from Strepsirrhine Primates	van Woerden JT, van Schaik CP, Isler K.	2010	Am Nat.
Energetic trade-offs between brain size and offspring production_ Marsupials confirm a general mammalian pattern	Isler K	2011	Bioassays
Environmental variation and the evolution of large brains in birds	Sayol F, Maspons J, Lapiedra O, Iwaniuk AN, Székely T, Sol D	2016	Nature Comm
Evolutionary Divergence in Brain Size between Migratory and Resident Birds	Sol D, Garcia N, Iwaniuk A, Davis K, Meade A, Boyle A, Székely T	2010	PloS One
Inter-Individual Variability in Fear of Humans and Relative Brain Size of the Species Are Related to Contemporary Urban Invasion in Birds	Carrete M, Tella JL.	2011	PloS One
Interspecific analysis of vehicle avoidance behavior in birds	Husby A, Husby M	2014	Behav Ecol
Large body and small brain and group sizes are associated with predator preferences for mammalian prey	Shultz S, Finlayson LV	2010	Behav Ecol
Large brains buffer energetic effects of seasonal habitats in cataeehine primates	van Woerden JT, Willems EP, van Schaik CP, Isler K.	2012	Evolution
Large Brains, Small Guts The Expensive Tissue Hypothesis Supported within Anurans	Liao WB, Lou SL, Zeng Y, Kotrschal A.	2016	Am Nat.
Larger brain size indirectly increases vulnerability to extinction in mammals	Gonzalez-Voyer A, González-Suárez M, Vilà C, Revilla E.	2016	Evolution
Life history costs and benefits of encephalization_ a comparative test using data from long-term studies of primates in the wild	Barrickmana NL, Bastiana ML, Isler K, Schaik CP	2008	J Human Evol
Maternal investment, life histories, and the costs of brain growth in mammals	Barton RA, Capellini I.	2011	Proc Natl Acad Sci
Mating system and brain size in bats	Pitnick S, Jones KE, Wilkinson GS	2006	Proc Biol Sci.
Sexual selection uncouples the evolution of brain and body size in pinnipeds	Fitzpatrick JL, Almbro M, Gonzalez-Voyer A, Hamada S, Pennington C, Scanlan J, Kolm N.	2012	J Evol Biol.
Social bonds in birds are associated with brain size and contingent on the correlated evolution of life-history and increased parental investment	Shultz S, Dunbar R.I.M	2010	Biol J Linn Soc
Social fishes and single mothers_ brain evolution in African	Gonzalez-Voyer A, Winberg S, Kolm N.	2009	Proc Biol Sci.
Sociality, ecology, and relative brain size in lemurs	MacLean EL, Barrickman NL, Johnson EM, Wall CE.	2009	J Hum Evol.
The evolution of hippocampus volume and brain size in relation to food hoarding in birds	Garamszegi LZ, Eens M	2004	Ecol Lett
The evolution of large brain size in birds is related to social, not genetic, monogamy	West RJD	2014	Biol J Linn Soc
The Evolution of Relative Brain Size in Marsupials Is Energetically Constrained but Not Driven by Behavioral Complexity	Weisbecker V, Blomberg S, Goldizen AW, Brown M, Fisher D.	2015	Brain Behav Evol.
Why do parasitic cuckoos have small brains: insights from evolutionary sequence analyses	Boerner M, Krüger O.	2008	Evolution
